# ESAT6-Induced IFNγ and CXCL9 Can Differentiate Severity of Tuberculosis

**DOI:** 10.1371/journal.pone.0005158

**Published:** 2009-04-02

**Authors:** Zahra Hasan, Bushra Jamil, Mussarat Ashraf, Muniba Islam, Muhammad S. Yusuf, Javaid A. Khan, Rabia Hussain

**Affiliations:** 1 Department of Pathology and Microbiology, The Aga Khan University, Karachi, Pakistan; 2 Department of Medicine, The Aga Khan University, Karachi, Pakistan; University of California Merced, United States of America

## Abstract

**Background:**

Protective responses against *Mycobacterium* tuberculosis are dependent on appropriate T cell and macrophage activation. Mycobacterial antigen six kDa early secreted antigenic target (ESAT6) and culture filtrate protein 10 (CFP10) can detect *M. tuberculosis* specific IFNγ responses. However, most studies have been performed in non-endemic regions and to study pulmonary tuberculosis (PTB). We have studied ESAT6 and CFP10 induced cytokine and chemokines responses in PTB and extrapulmonary (EPul) TB.

**Methodology:**

IFNγ, IL10, CXCL9 and CCL2 responses were determined using an *ex vivo* whole blood assay system in PTB (n = 30) and EPulTB patients with limited (LNTB, n = 24) or severe (SevTB, n = 22) disease, and in healthy endemic controls (ECs). Responses to bacterial LPS were also determined.

**Principal Findings:**

ESAT6- and CFP10-induced IFNγ was comparable between ECs and TB patients. Both ESAT6- and CFP10-induced IFNγ secretion was greater in LNTB than PTB. ESAT6-induced CXCL9 was greater in EPulTB as compared with PTB, with an increase in SevTB as compared with LNTB. CFP10-induced CCL2 was higher in PTB than LNTB patients. LPS-stimulated CXCL9 was greatest in SevTB and LPS-induced CCL2 was increased in PTB as compared with LNTB patients. A positive correlation between ESAT6-induced IFNγ and CXCL9 was present in all TB patients, but IFNγ and CCL2 was only correlated in LNTB. ESAT-induced CCL2 and CXCL9 were significantly associated in LNTB while correlation in response to LPS was only present in SevTB.

**Conclusions:**

ESAT6 induced IFNγ and CXCL9 can differentiate between limited and severe TB infections.

## Introduction

Protective immunity against *Mycobacterium tuberculosis* is dependent on the interplay between activated T cells, macrophages and other leucocytes, proinflammatory cytokines such as, interferon gamma (IFNγ), tumor necrosis factor-alpha (TNFα) and interleukin (IL)-12, and down-modulatory cytokines IL-10 and transforming growth factor beta (TGF)-β [1], [2]. IL10 produced by macrophages is important in regulating the Th1 cytokine balance and down regulates proinflammatory responses [3]. TNFα regulated granuloma formation relies on effective recruitment of leucocytes, dependent on chemokines such as the CC chemokines; CCL2, CCL3, CCL4, CCL5 and CXC chemokines; CXCL8, CXCL9 and CXCL10 [4]–[7]. Coordinate expression of these chemokines with their receptors is essential for granuloma formation. Deficiency of the CCL2 receptor, CCR2, has been shown to result in progressive infection by *M. tuberculosis* in mice [8]. CXCL9 stimulates T lymphocytes [9] and has previously been shown to be a sensitive predictive marker for antigen-specific IFNγ production and IFNγ secreting cells [10].

Rapid diagnosis of tuberculosis (TB) infections is essential for early detection and control of disease. Six kDa early secreted antigenic target (ESAT6) and culture filtrate protein 10 (CFP10) are both encoded by the region of difference 1 (RD1) which is present in *M. tuberculosis* and *M. bovis,* but absent from *M. bovis* BCG and most environmental mycobacteria [11], [12]. ESAT6 is a immunodominant T cell-stimulatory antigen and is recognized by specific IFNγ-secreting T cells present in greater numbers in patients with active disease as compared with those who are un-infected [13], [14]. Commercial tests employing IFNγ release assays (IGRA) such as, ESAT6 Quantiferon TB-2G [15], [16] and T-SPOT TB are increasingly available as diagnostic and predictive tests for vaccination. Therefore, ESAT6 and CFP10 induced IFNγ responses have been shown to be useful in discriminating infected individuals from healthy controls [17]–[19]. However, there are controversial reports from endemic regions. In TB endemic regions, IFNγ can be modulated by natural exposure to *M. tuberculosis* or non-tuberculous mycobacteria as well as BCG vaccination [20], [21]. Recent studies have shown that the sensitivity of IGRA may be related to the ethnic origin and age of the individual tested [22]. The sensitivity of ESAT6 and CFP10 induced *Mycobacterium*-specific T cell responses is greatest in a BCG unvaccinated population in a non-endemic region and most studies have been performed in areas of low tuberculosis (TB) transmission [23], with less data available from high transmission TB endemic regions [24]. A recent study has shown that the utility of using additional biomarkers such as CCL2 and CXCL10 together with IFNγ to assess mycobacterial antigen induced responses in identifying patients with *M. tuberculosis* infection [25]. However, most research has been performed on patients with pulmonary tuberculosis (PTB). Although PTB is the predominant form of tuberculosis, extra-pulmonary disease (EPulTB) involving; lymph nodes, skeletal system, abdomen, meningeal, miliary or disseminated forms of the disease remain common [26], [27]. EPulTB presents an additional burden due to the problems associated with diagnosis and treatment of infections in extra-pulmonary sites. It is therefore essential to assess IFNγ induced responses to mycobacterial antigens in endemic areas and in patients with PTB and EPulTB. This work has been carried out in Pakistan which is ranked 8^th^ amongst high TB burden countries and has an incidence of 181/100,000 cases/year, where 44% pulmonary and 15% extrapulmonary samples are listed under new case detection for DOTS [28].

The *ex vivo* whole blood assay model is useful in assessing host immune responses in tuberculosis patients [29] [30], despite its limitations in identifying the cellular sources of secreted products. We have investigated mycobacterium specific responses using antigens ESAT6 and CFP10 and also response to bacterial lipopolysaccharide (LPS) in whole blood cultures of patients with PTB (n = 30) and EPulTB (n = 46) with differing disease severity. Cytokines (IFNγ and IL10) and chemokines (CXCL9 and CCL2) were measured in each donor. Our results indicate that ESAT6-induced IFNγ and CXCL9 responses do not discriminate between active TB patients and healthy endemic controls (ECs), but that they do differentiate severity of infections.

## Results

### Characteristics of study group

Table 1 illustrates hematological characteristics of patients in the study. EPulTB patients were divided according to WHO ranking of severity into less severe TB (LNTB) or severe TB (SevTB) [28], see methods and Table 2. The study groups were comparable with respect to age and gender. As expected [27], erythrocyte sedimentation rate (ESR) was found to be raised in TB patients as compared with ECs; PTB, LNTB and SevTB (p<0.01, 0.01, 0.01, respectively). Total leucocyte (TLC) and monocyte counts were comparable between patients and controls. However, in PTB and SevTB, lymphocyte counts were decreased (p = 0.002, p<0.01, respectively), while neutrophil counts were increased (p = 0.02, p = 0.008, respectively).

**Table 1:** Characteristics of tuberculosis patients and healthy endemic controls.

Characteristic	EC	PTB	LNTB	SevTB
**Number**	17	30	24	22
**Age (y)**	27.5±5.9	27.8±12.2	33.2±12.4	36.68±18.7
**Male∶Female**	9 vs 8	11 vs 19	9 vs 15	10 vs 12
**ESR (mm/h)**	5.4±2.9	54±37*	34.4±25.2*	45.4±33.8*
**Hb (g/dL)**	13.6±1.1	11.8±1.8*	12.3±1.6*	12.2±1.5*
**TLC (10e^9^/L)**	7.4±1.6	9.4±5.1	7.7±2.3	8.1±2.8
**Lymphocytes (10e^9^/L)**	2.3±0.5	1.7±0.7*	2.0±0.7	1.3±0.4*
**Monocytes (10e^8^/L)**	5.3±1.6	6.0±2.8	5.8±2.4	4.7±1.7
**Neutrophils (10e^9^/L)**	4.3±1.2	7.0±4.7*	5.0±2.0	6.0±2.7*

EC, healthy endemic controls; PTB, pulmonary TB; LNTB, limited extrapulmonary TB; SevTB, severe extrapulmonary TB.

* denotes significant difference (p<0.05) as compared with EC.

**Table 2:** Diagnostic criteria for patients with severe extrapulmonary tuberculosis.

No.	Site of tuberculosis	Abscess	Microscopy^a^	Radiology^b^	AFBC^c^	Histopathology^d^
1	Spine	Yes		Yes		Positive
2	Spine	Yes		Yes	Negative	Positive
3	Spine	Yes		Yes		
4	Spine	Yes		Yes	Positive	Positive
5	Spine	Yes		Yes		
6	Spine	Yes		Yes		
7	Spine	Yes		Yes		
8	Spine		Negative	Yes	Positive	Positive
9	Spine	Yes	Positive			Positive
10	Meninges^e^			Yes	Negative	
11	Meninges^e^			Yes	Negative	
12	Meninges		Positive	Yes	Positive	Positive
13	Meninges		Positive	Yes	Positive	
14	Meninges^e^		Negative	Yes	Negative	
15	Meninges^e^					Positive
16	Abdomen		Positive	Yes	Negative	
17	Abdomen		Negative			Positive
18	Abdomen			Yes	Negative	
19	Miliary			Yes	Negative	Positive
20	Intestines			Yes		Positive
21	Bilateral Pleural		Negative	Yes	Negative	Positive
22	Bilateral Pleural			Yes		

a indicates acid fast bacilli staining of smears.

b includes Xray, MRI or CT imaging characteristic of tuberculosis.

c acid fast bacilli culture using BACTEC radiometric assay, Becton Dickinson, USA.

d biopsy results indicate caseating or necrotic granulomatous inflammation indicative of *M. tuberculosis* infection.

e showed a favorable clinical response to anti-tuberculous treatment.

### Cytokine and chemokine secretion in unstimulated whole blood cultures of TB patients and endemic controls

IFNγ, IL10 and CXCL9 and CCL2 secretion from unstimulated cells was measured in healthy endemic controls (ECs, n = 17) and in TB patients (n = 76, Table 3). IFNγ, IL10 and CCL2 secretion from unstimulated whole blood cells of ECs and TB patients were comparable. However, CXCL9 secretion from unstimulated whole blood cells of ECs was greater than in TB patients (p = 0.031).

**Table 3:** ESAT6- and CFP10-induced cytokine and chemokine responses.

**Unstimulated cells**				
Group (n)	IFNγ (Mean±SD pg/ml)	IL10 (Mean±SD (pg/ml)	CXCL9 (Mean±SD pg/ml)	CCL2 (Mean±SD pg/ml)
EC (17)	2.0±2.8	136.8±287	7.9±20.2	86±327
TB (76)	6.3±13	10.8±17.9	2.3±18.8*	90.4±334
**ESAT6-induced responses**				
Group (n)	δIFNγ (Mean±SD pg/ml)	δIL10 (Mean±SD (pg/ml)	δCXCL9 (Mean±SD pg/ml)	δCCL2 (Mean±SD pg/ml)
EC (17)	29±46	12±36	1.7±6.5	1456±1300
TB (76)	299±520	93±225	123±278*	1197±843
**CFP-induced responses**				
Group (n)	δIFNγ (Mean±SD pg/ml)	δIL10 (Mean±SD (pg/ml)	δCXCL9 (Mean±SD pg/ml)	δCCL2 (Mean±SD pg/ml
EC (17)	286±442	23.4±70.2	19.6±31.9	2787±2293
TB (64)	635±720	155±237*	792±1113*	2076±1149
**LPS-induced responses**				
Group (n)		δIL10 (Mean±SD (pg/ml)	δCXCL9 (Mean±SD pg/ml)	δCCL2 (Mean±SD pg/ml
EC (17)		342±577	17.3±46	3536±1504
TB (64)		614.4±733	390±979*	1920±1047*

EC, healthy endemic controls; TB, tuberculosis patients.

‘δ’ denotes cytokine secretion after background subtraction in each case.

* denotes significant difference (p<0.05) as compared with EC.

All statistical analyses performed using the Mann-Whitney U non-parametric test.

### Mycobacterial antigens ESAT6-induced IFNγ, IL10, CCL2 and CXCL9 in TB patients

ESAT6–induced cytokine and chemokines responses were determined in whole blood cells of 76 TB patients. Upon stimulation by ESAT6, the trend of IFNγ secretion at 5 day post-stimulation was greater in TB patients as compared with ECs but did not achieve statistical significance (Table 3). ESAT6-induced IL10 secretion was also increased in TB patients but was not statistically significant as compared with ECs (p = 0.07).

CXCL9 has been shown to be induced by ESAT6 in patients with pulmonary TB [17]. We found ESAT6-induced CXCL9 to be significantly greater in TB patients as compared with ECs (p = 0.003). While, no difference was observed between ESAT6-induced CCL2 in TB patients and ECs.

### CFP-10 induced cytokine and chemokine responses

We also compared CFP10 induced whole blood cell responses in ECs and TB patients. CFP10-induced IFNγ was increased in TB as compared with ECs but was not significantly different (Table 3). CFP10-induced IL10 (p = 0.002) secretion and also CXCL9 (p = 0.001) secretion was significantly raised in TB patients. However, there was no difference in CFP10-induced CCL2 between patients and controls. Although the trend of responses to ESAT6 and CFP10 were comparable, the magnitude of cytokine responses to CFP10 was approximately 2 fold greater than that induced by ESAT6.

### LPS-induced cytokine and chemokine secretion in TB patients

To investigate whether the differences in cytokine secretion observed were specific to mycobacterial antigens, we also studied cytokine and chemokine responses to bacterial lipopolysaccharide (LPS), a strong activator of macrophages. LPS-induced IL10 was not different between TB patients and ECs, Table 3. However, LPS-induced CXCL9 was significantly greater (p = 0.008), while CCL2 was reduced (p<0.01) in TB patients as compared with ECs.

### Differential ESAT6-induced IFNγ in pulmonary and extrapulmonary TB

We further investigated IFNγ responses to mycobacterial antigens in TB patients with pulmonary and extrapulmonary disease of differing severity. Pulmonary TB patients included in the study comprised those with minimal (n = 6) and moderate (n = 24) disease. However, we found antigen stimulated IFNγ secretion in minimal and moderate PTB patients to be comparable between groups (data not shown) and have therefore performed further analysis on the combined minimal/ moderate PTB group.

**Figure 1:**
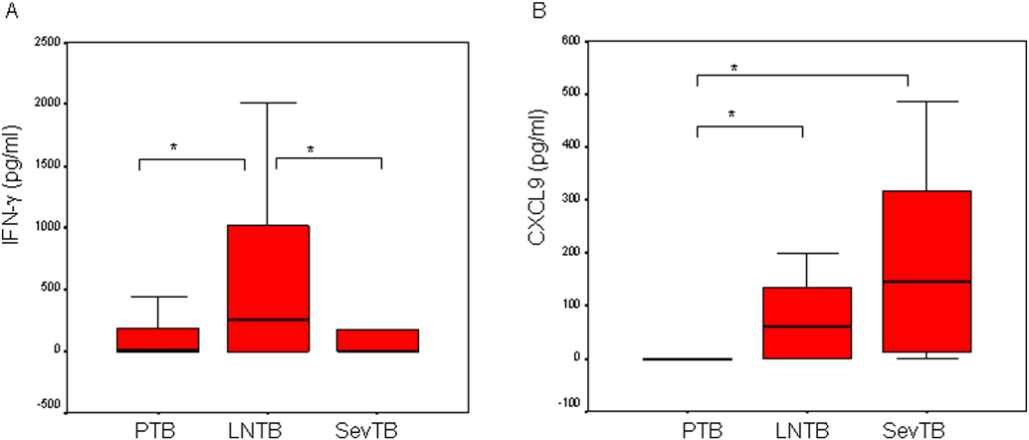
Differential ESAT6-induced IFNγ and CXCL9 in pulmonary and extrapulmonary TB. Whole blood cells were stimulated with ESAT6 at 5 µg/ml, and IFNγ measured in cell supernatants harvested at 5 day post-stimulation, while CXCL9 was measured at 2 days post-stimulation. TB patient groups comprised those with pulmonary TB (PTB, n = 30), less severe EPulTB (LNTB, n = 24) and severe EPulTB (SevTB, n = 22). The box plots represent data from each group after cytokine secretion from unstimulated cells has been subtracted. The whiskers indicate the 25^th^ and 75^th^ quartile respectively, while the median line separates the two. ‘*’ denotes significant differences between groups, p<0.05; (*). A. IFNγ, B. CXCL9.

Responses in PTB patients were compared with those of EPul-TB; limited (LNTB) or severe (SevTB). As illustrated in Fig. 1A, ESAT6-induced IFNγ in LNTB (median, 253 pg/ml) was significantly greater than in PTB (median, 3 pg/ml, p = 0.011). IFNγ responses in LNTB were significantly greater as compared with SevTB (median, 1 pg/ml, p = 0.029). ESAT6-induced IL10 was negligible in all TB groups (PTB, LNTB, SevTB; 0, 3, 3 pg/ml respectively, data not shown).

The balance between proinflammatory IFNγ and downmodulatory IL10 has been shown to be critical in determining outcome of mycobacterial infections [31], and the IFNγ (5d) /IL10 (2d) ratio induced in response to mycobacterial antigens has been shown to be to be an indicator of disease severity in tuberculosis [32]. We found the ESAT6-induced IFNγ/IL10 ratio to be significantly greater in LNTB (median, 3.5) as compared with the SevTB group (median, 1; p = 0.011, data not shown), consistent with these earlier reports.

ESAT6-induced CXCL9 was significantly raised in EPulTB groups SevTB (p<0.01) and LNTB (p = 0.029) as compared with PTB (Fig. 1B). No difference was observed between ESAT6-induced CCL2 in whole blood cells of PTB, LNTB or SevTB patients (median; 782, 1568 and 1056 pg/ml, respectively, data not shown).

### CFP10-induced cytokine and chemokine responses in PTB and EPulTB

**Figure 2:**
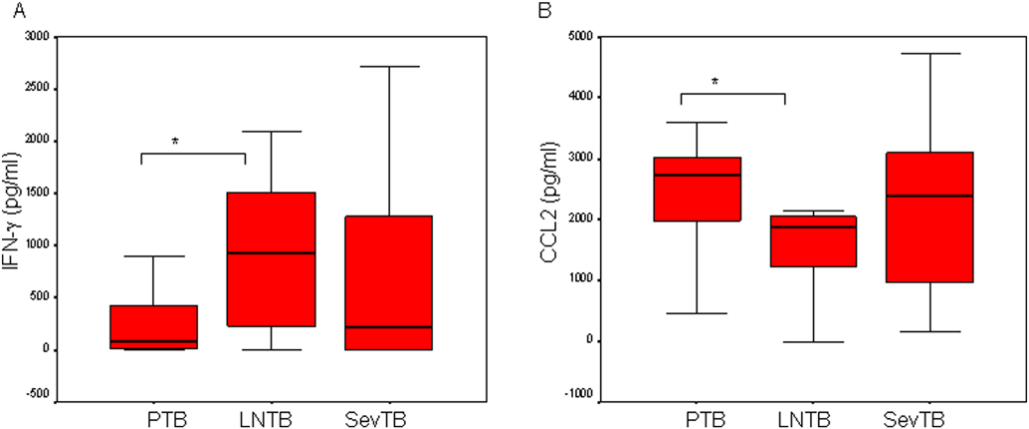
Differential CFP10-induced IFNγ and CCL2 in PTB and EPulTB. Whole blood cells from different patients were stimulated with CFP10 at 5 µg/ml. IFNγ and CCL2 were measured in cell supernatants. All other parameters as described in Fig. 1. The box plots indicate cytokine secretion in PTB, LNTB and SevTB groups. A. IFNγ, B. CCL2.

CFP10-induced IFNγ was greater in LNTB as compared with PTB patients (p = 0.044), but did not differ from with SevTB, Fig. 2A. CFP10-induced IL10 was found to be comparable between the TB patient groups (median; PTB, 22; LNTB, 30; SevTB, 52 pg/ml, data not shown).

Coincident with results observed in response to ESAT6, CFP10-induced IFNγ /IL10 ratio was also greater in LNTB patients (median, 35) as compared with those with SevTB (median, 0.1; p = 0.001, data not shown).

No differences were observed in CFP10-induced CXCL9 in PTB (51 pg/ml) and EPulTB groups, although CFP10 induced a greater trend of CXCL9 secretion in LNTB and SevTB (543, 192 pg/ml, respectively, data not shown). As observed in the case of IFNγ, CFP10-induced CXCL9 was 2 fold greater in magnitude as compared with that in response to ESAT6.

However, CFP10-induced a significantly greater CCL2 response in PTB patients as compared with LNTB (p = 0.036, Fig. 2B).

### LPS-induced CXCL9 and CCL2 is greater in severe as compared with limited TB

**Figure 3:**
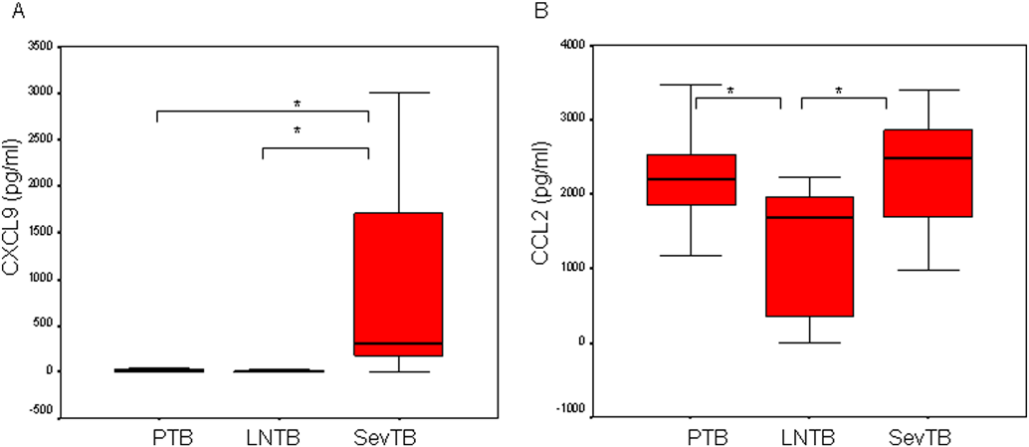
Differential LPS-induced CXCL9 and CCL2 in limited and severe TB. Whole blood cells were stimulated with LPS at 1 µg/ml and CXCL9 measured in cell supernatants harvested at 2 days post-stimulation. All other parameters as described in Fig. 1. A. CXCL9 and B. CCL2.

When LPS-induced CXCL9 was measured in PTB, LNTB and SevTB groups it was observed that chemokine levels were significantly greater in SevTB as compared with PTB and LNTB (p = 0.001, 0.004, respectively), Fig. 3A. In contrast, LPS-induced CCL2 was greater in PTB as compared with LNTB (p = 0.01) and also in SevTB patients as compared with LNTB (p = 0.003), Fig. 3B.

### Relationship between CXCL9, CCL2 and IFNγ secretion in tuberculosis patients

We next determined the association between cytokine and chemokine responses induced to mycobacterial antigen ESAT6 and to LPS in different TB patient groups. A Spearman's rank correlation analysis showed ESAT6-induced secretion of IFNγ and CXCL9 to be positively associated in all TB groups (Table 4). A significant association between ESAT6-induced IFNγ and CCL2 (R = 0.471, p = 0.027) and between CCL2 and CXCL9 (R = 0.57, p = 0.006) was observed only in the LNTB group, data not shown.

**Table 4:** Correlation between cytokines and chemokines stimulated by ESAT6 and LPS.

	IFNγ to CXCL9		
	TB group		
	**PTB**	**LNTB**	**SevTB**
**ESAT6**			
R	0.583*	0.508*	0.577*
*p-value*	*0.001*	*0.016*	*0.015*

	CXCL9 to CCL2		
	TB group		
	**PTB**	**LNTB**	**SevTB**
**LPS**			
R	−0.073	0.16	0.689**
*p-value*	*0.789*	*0.569*	*0.006*

EC, endemic controls; PTB, pulmonary TB; LNTB, less severe EPul-TB; SevTB, severe EPul-TB.

R values 0.4–0.6 indicate a moderate positive association.

* while 0.6–0.8 indicate a strong positive association

** p value≤0.05 are significantly different.

No association was found between LPS-induced IL10 and either CXCL9 or CCL2. However, LPS-induced CXCL9 and CCL2 were found to be positively associated in patients with SevTB but not in PTB or LNTB groups (Table 4).

## Discussion

The relationship between cytokines, IFNγ and IL10 in regulating the outcome of *M. tuberculosis* infections is well studied. We have recently shown that circulating levels of IFNγ and CXCL9 differ in patients with pulmonary and extrapulmonary disease [33]. We show here that the relationship between mycobacterial antigen induced IFNγ and CXCL9 may play a role in determining disease severity in tuberculosis.

Our study illustrates that in a TB endemic region, ESAT6-induced IFNγ responses may not be as effective in distinguishing patients with active disease from healthy individuals in endemic regions. This may be attributed to immune modulation of IFNγ responses to specific *M. tuberculosis* antigens as has been shown in a recent study of household contacts of TB patients [31]. Also, a high rate of transmission in endemic regions may result in modulation of IFNγ and IL10 responses due to increased exposure to environmental mycobacteria [21], [34].

Amongst healthy controls we studied asymptomatic individuals who were both TST− and TST+. However, we did not observe any difference between cytokine or chemokine induced responses from whole blood cells of either group either after measurement from unstimulated cells or from ESAT6 or CFP10 stimulated cells (data not shown), corresponding with previous reports [35]. However, when both EC groups; TST− and TST+ donors were compared with TB patients, a significant differences in ESAT6- and CFP10-induced CXCL9 and CCL2 secretion was found in TB patients as compared with TST− individuals but not in the TST+ group (data not shown). This may be attributed to latent infection in the TST+ controls which would raise specific cytokine responses to mycobacterial antigens. Previous reports have shown Th1-like effector memory cells to be increased in bronchoalveolar cells from TST+ individuals as compared with TST− individuals, with coordinate increase in IFNγ and IFNγ regulated chemokines [36]. Therefore, in our study we have compared responses of TB patients with Ecs who are TST− in order to have the least exposed group as a control.

In all cases, antigen-induced IFNγ secretion increased in magnitude from 2 to 5 days post-stimulation (data not shown). This may be attributed to the differing cellular sources of IFNγ whereby at the earlier time point there would be a greater contribution from effector T cells, NK cells, γδ T cells and activated macrophages, while at the latter time point the IFNγ may primarily be released from mycobacterium specific T memory cells.

We found ESAT6-induced IFNγ response to be decreased in patients with severe disseminated TB, comprising of those with spinal, meningeal, abdominal, miliary involvement, as compared with localised disease. Tuberculous lymphadenitis is the most common presentation of extrapulmonary TB. It is often a self-limiting disease and is less severe as compared with other forms. Increased IFNγ responses in less severe TB infections correlates with previous work which has shown the frequency of antigen specific CD4 T cells to be greater in latently infected individuals and in those with minimal disease with a low bacterial burden as compared with patients with a higher bacterial load [37]. Therefore, patients with limited extrapulmonary disease may be more likely to have an increased number of IFNγ secreting T cells as compared with those with progressive disease [37]. Previous reports haves shown that in pulmonary TB, there is a decrease in IFNγ responses related to increased severity of disease [38], [39].

Our data corresponds with previous work which has shown that differences in the severity of extrapulmonary TB can be defined by the IFNγ: IL10 ratio to mycobacterial antigens, with a direct relationship between disseminated disease or disease localized to peripheral sites without lung involvement [32], [40].

CXCL9 is IFNγ induced and has been shown to be an early marker of IFNγ activation and of T cells secreting IFNγ [10]. CXCL9 is a chemotactic ligand produced by macrophages and other antigen presenting cells at the site of inflammation [9]. We found ESAT6-induced CXCL9 to be increased in TB patients, corresponding with previous reports [17]. This corresponds with reports by Gonzalez-Juarrero *et* al. who show upregulated expression of IFNγ regulated chemokines transcripts in response to *M. tuberculosis* coordinate with inflammation in the mouse model [41]. The increase in CXCL9 observed in severe extrapulmonary disease corresponds with literature which have shown chemokines responses to be increased in severe TB such as in tuberculous meningitis [42], [43]. CFP10-induced activation of CXCL9 was greater than that observed for ESAT6 just as observed in the case of IFNγ. Although the trend of responses to ESAT6 and CFP10 was comparable, ESAT6 appeared to elicit more significant difference in responses between limited and severe TB. This may be due to differential recognition of cell surface receptors for the recombinant antigens on T cells which may determine the magnitude of IFNγ response and subsequently impact macrophage activation.

ESAT6 stimulated secretion of induced- CCL2 was not different between TB groups, further indicating the utility of this antigen in eliciting T cell specific responses.

LPS is a strong stimulant of monocyte responses, and both CXCL9 and CCL2 are produced by monocytes. Raised LPS-induced CXCL9 in severe extrapulmonary TB may be indicative of the increased inflammatory processes ongoing in progressive, disseminated disease and an absence of regulation. CCL2 has previously been shown to be increased in PTB patients as compared with Epul-TB patients and our results with LPS support this. In addition, we observe that as in case with CXCL9, CCL2 levels are raised in severe TB as compared with limited disease in patients with extrapulmonary TB.

Our correlation analysis between the cytokines and chemokines produced by whole blood cells of TB patients indicates differential regulation of cytokines in response to mycobacterial antigen stimulation in patients with differing disease severity. A positive association was found between ESAT6 induced IFNγ and CXCL9 in all TB patients indicating that this balance may play a predictive role in the Th1-like cytokine balance required for protection against *M. tuberculosis.* However, as association between the macrophage activating CCL2 and the T cell activation IFNγ and CXCL9 was only found in LNTB patients. When LPS-induced responses were investigated, there appeared to be no association between CCL2 and CXCL9 in limited disease (PTB and LNTB), but a correlation between these two chemokines was present in severe extrapulmonary TB. It is possible that in severe extrapulmonary disease where there is an overall reduction in IFNγ possibly due to T cell anergy, CXCL9 secretion may lead to increased macrophage recruitment and inflammation, the lack of association between the chemokines and T cell regulation may result in progressive disease related pathology in the host. Overall, this work proposes differential chemokine regulation in limited and severe tuberculosis infections which may explain subsequent pathophysiological changes in the host. Further investigations into the mechanisms responsible for the control of macrophage activation in different disease states may further explain this phenomenon.

## Materials and Methods

### Ethics statement

This study was conducted according to the principles expressed in the Declaration of Helsinki. The study was approved by the Institutional Review Board of Aga Khan University hospitals. All patients provided written informed consent for the collection of samples and subsequent analysis.

### Subject selection

Seventy six patients were recruited from the Aga Khan University Hospital and Medical College (AKUH) and Masoomeen Hospital, Karachi. All study subjects were examined and evaluated by infectious diseases consultants. Patients were newly diagnosed or had taken less than 7 days of anti-tuberculous therapy (ATT). Patients with significant co-morbid conditions including diabetes mellitus, chronic renal failure, chronic liver disease, patients on high dose corticosteroid therapy were excluded to assure relatively unmodulated immunological parameters. All patients were tested and found to be HIV negative.

Patients with pulmonary TB (PTB, n = 30) were diagnosed by clinical examination, chest X-ray, sputum acid fast bacillus (AFB) Zeihl Neelson staining, AFB culture and/ or clinical response to treatment (along with fever, cough and weight loss). All PTB patients had findings consistent with active TB as evaluated by one of the consulting physicians. Fifteen patients were found to have a positive AFB smear microscopy. Nine PTB patients had an AFB negative smear, but showed a favourable clinical response to ATT. Patients were diagnosed as having minimal, moderate or advanced pulmonary tuberculosis using a modified classification of the National Tuberculosis Association of the USA based on extent of lung tissue involvement [44], [45]. Of the PTB patients 6 had minimal, while 24 had moderate disease.

Diagnosis of TB lymphadenitis, spinal, abdominal and genitourinary TB was based on histopathological findings of chronic granulomatous inflammation with caseous necrosis, AFB staining and culture and supportive radiological evidence on CT or MRI. Diagnosis of meningeal TB was based on CSF biochemical findings, supported by AFB culture and findings on contrast-enhanced CT and/or MRI. Pleural TB was diagnosed on the basis of pleural fluid biochemical findings, AFB culture, histopathological findings on pleural biopsy and supportive radiological evidence on X-rays and/or contrast-enhanced CT scan

Patients with extrapulmonary TB (Epul-TB) were divided into two groups according to the WHO ranking of diseases severity based on bacillary load, extent of disease and anatomical site [28]. Epul-TB patients were subsequently classified into less severe (LNTB) or severe (SevTB) disease. Of the 24 patients with LNTB, 23 had lymphadenitis and 1 had genitourinary TB. All LNTB patients were confirmed on histological findings consistent with tuberculosis. Twenty two patients had SevTB and their diagnostic criteria are provided in Table 2.

BCG-vaccinated asymptomatic healthy volunteers who were staff at AKU with no known exposure to TB were used as endemic controls (Ecs). All volunteers had a normal chest X-Ray. Tuberculin skin testing (TST) was assessed by administering five tuberculin units on the volar surface of the right arm subcutaneously, and read by a single reader at 48 h. An induration of ≥10 mm was used as a cutoff for positive responses and TST− (n = 17) Ecs were included in the study.

### Reagents

Recombinant antigens ESAT6 and CFP10 were provided through the TB Vaccine Testing and Research Materials Contract, NIH, NIAID NO1-A1-40091, awarded to Colorado State University, USA.

### Whole blood assay

Venous blood was diluted 1∶10 in RPMI-1640 medium and 200 µl set up per well in a 96 well tissue culture plate as described previously [29]. Whole blood cells were stimulated with recombinant antigens ESAT-6 (5 µg/ml) and CFP-10 (5 µg/ml) and cultured for up to 5 days. All samples were set up in replicates. Supernatants were collected at 2 and 5 day post-stimulation for cytokine and chemokine measurements. Samples were centrifuged to collect any cellular debris, aliquoted and stored at –70 C until tested. Cytokines were measured at both 2 and 5 days post-stimulation. We have presented IFNγ responses from whole blood cells at 5 day of culture to focus on T cell responses [21], [29]. IL10 increased only marginally from 2 to 5 day post-stimulation and there was no significant increase between the two times studied (data not shown). Therefore, we have further analyzed IL10 responses at 2 day post-stimulation. CCL2 and CXCL9 were optimally measured at 2 days post-stimulation.

### ELISA for IFNγ, IL-10 and CXCL9

IFNγ and IL-10 was detected in cellular supernatants by using standards and ELISA reagents obtained from Endogen (Rockford, IL, USA). Cytokines were measured using a sandwich ELISA technique according to the manufacturer’s instructions and as reported previously [30]. Recombinant human cytokine was used to obtain a dose response curve with a range of detection from 3.9–1000 pg/ml. All experimental samples were tested in duplicate.

CXCL9 and CCL2 standards and monoclonal antibody pairs for capture and detection were obtained from R&D Systems (Abingdon, UK). All measurements were carried out according to the manufacturer’s recommendations and as described previously [30]. Recombinant human cytokine was used to obtain a dose response curve with a range of detection from 6.25–500 pg/ml for CXCL9 and 6.25–1000 pg/ml for CCL2.

### Statistical analysis

Data is represented by box blots illustrating 25^th^ and 75^th^ quartiles with the median shown as a horizontal bar. Non-parametric statistical analysis was performed using Kruskal-Wallis and Mann-Whitney U tests as required. P values≤0.05 were considered to be statistically significant. Spearman’s Rank Correlation was also performed, all using the Statistical Package for Social Sciences software (SPSS).
